# Preoperatively predicting early response of HCC to TACE using clinical indicators and MRI features

**DOI:** 10.1186/s12880-022-00900-8

**Published:** 2022-10-07

**Authors:** Zhi-Wei Li, A-Hong Ren, Da-Wei Yang, Hui Xu, Jian Wei, Chun-Wang Yuan, Zhen-Chang Wang, Zheng-Han Yang

**Affiliations:** 1 Department of Radiology, Beijing Friendship Hospital, Capital Medical University, 95 YongAn Road, Xicheng District, Beijing, 100050 People’s Republic of China; 2 Department of Interventional Radiography, Beijing Friendship Hospital, Capital Medical University, 95 YongAn Road, Xicheng District, Beijing, 100050 People’s Republic of China; 3 Center of Interventional Oncology and Liver Diseases, Beijing Youan Hospital, Capital Medical University, No.8 Xitoutiao, Youwai St, Fengtai District, Beijing, 100069 People’s Republic of China

**Keywords:** Hepatocellular carcinoma (HCC), Transarterial chemoembolization **(**TACE), Magnetic resonance imaging (MRI), Modified response evaluation criteria in solid tumors (mRECIST), Predictive factors

## Abstract

**Background:**

We aimed to evaluate the value of using preoperative magnetic resonance imaging (MRI) features and clinical indicators to predict the early response of hepatocellular carcinoma (HCC) to transcatheter arterial chemoembolization (TACE). We also aimed to establish a preoperative prediction model.

**Methods:**

We retrospectively reviewed data of 111 patients with HCC who underwent magnetic resonance imaging (MRI) before the first TACE and underwent MRI or computed tomography between 30 and 60 days after TACE. We used the modified response evaluation criteria in solid tumors for evaluating the TACE response. We used univariate and multivariate logistic regression analyses to identify independent predictors based on MRI features and clinical indicators. Moreover, receiver operating characteristic (ROC) curve analyses were performed to assess the diagnostic performance of the prediction model and each independent predictor.

**Results:**

Among the 111 included patients, 85 were men (76.6%). Patient age was 31–86 years (average age, 61.08 ± 11.50 years). After the first treatment session, 56/111 (50.5%) patients showed an objective response (complete response + partial response), whereas the remaining showed non-response (stable disease + local progressive disease). In the univariate analysis, we identified irregular margins, number of nodules, and satellite nodules as predictors of early objective response. However, in the multivariate logistic regression analysis, irregular margins, number of nodules and pretreatment platelet were identified as the independent predictors of early objective response. A combined prediction model was then established, which factored in irregular margins, the number of nodules, and the pretreatment platelet count. This model showed good diagnostic performance (area under the ROC curve = 0.755), with the sensitivity, specificity, positive predictive value, and negative predictive value being 78.6%, 69.1%, 72.1%, and 76.0%, respectively.

**Conclusions:**

Irregular margins, the number of nodules and the pretreatment platelet count are independent predictors of the early response of HCC to TACE. Our clinical combined model can provide a superior predictive power to a single indicator.

**Supplementary Information:**

The online version contains supplementary material available at 10.1186/s12880-022-00900-8.

## Introduction

At present, hepatocellular carcinoma (HCC) is the third most common cause of cancer-related deaths and the fifth most common cancer worldwide according to the World Health Organization estimates [1]. Because of the high incidence of hepatitis B in China, chronic hepatitis B virus infection is considered a major cause of HCC [2]. For unresectable HCC, particularly for Barcelona Clinic Liver Cancer (BCLC) classification stage B (intermediate stage) HCC, transcatheter arterial chemoembolization (TACE) is widely acknowledged as a standard and routine treatment approach [3]. However, the BCLC system has a major limitation. Within a single BCLC stage, the system allows for the inclusion of a significantly heterogeneous patient population that may substantially vary in terms of liver functional reserve, disease etiology, and the degree of tumor extension. Therefore, even within a single BCLC stage, there is marked heterogeneity in the therapeutic efficacy [4]. Consequently, among HCC patients treated with TACE, up to 60% reportedly do not benefit from the treatment, thus warranting further intervention using alternative therapies [5]. Thus, preoperatively predicting the treatment response after TACE in patients with HCC would greatly improve patient prognosis and guide therapeutic strategies.

Thus far, several studies have investigated the prognosis of HCC patients after TACE. Previous studies have reported that tumor location in segments 1 and 4 is associated with reduced chances of complete response, whereas a tumor size < 5 cm is a positive predictor of a complete response [6]. Zhang et al. [7] suggested that the early response of HCC to TACE can be predicted by the following attributes: irregular margins for tumors measuring 2–5 cm and irregular margins, abnormal AFP levels, and arterial peritumoral enhancement for tumors measuring > 5 cm. Furthermore, the overall response to TACE can also be predicted by the number of tumors present [8]. Some studies have found that the results of functional magnetic resonance imaging (fMRI) can help predict early responses in patients with HCC with satisfactory accuracy. In particular, when using diffusion-weighted imaging performed 1 month after undergoing TACE for HCC, the apparent diffusion coefficient ratio is reportedly an independent predictor of progression-free survival [9]. Additionally, other emerging fMRI techniques, such as diffusion kurtosis imaging and intravoxel incoherent motion, are also helpful in assessing early treatment response to TACE [10, 11]. Moreover, several inflammatory markers are associated with the postoperative prognosis of HCC. Schobert et al. reported that the neutrophil-to-lymphocyte ratio (NLR) and platelet-to-lymphocyte ratio (PLR) are tumor response predictors in patients with HCC having undergone drug-eluting bead transcatheter arterial chemoembolization (DEB-TACE) [12]. Wang et al. suggest that in HCC patients undergoing TACE, the pretreatment platelet count is a useful biomarker for predicting the prognostic outcomes [13].

However, image acquisition using fMRI takes a long time and is influenced by several aspects, such as the MR sequence type, respiratory motion, and scan parameters, thus limiting its wide applicability in clinical work. Therefore, the prediction of tumor responses after TACE for HCC is undertaken using conventional MRI features. Furthermore, to our knowledge, the combined use of MRI and clinical indicators remains relatively less in this regard. Thus, our study aimed to evaluate the combined value of MRI features and preoperative clinical indicators for predicting the early response of HCC to TACE. Moreover, we also aimed to create a preoperative prediction model that can improve patient prognosis by guiding therapeutic strategies.

## Materials and methods

### Patient information

**Fig. 1:**
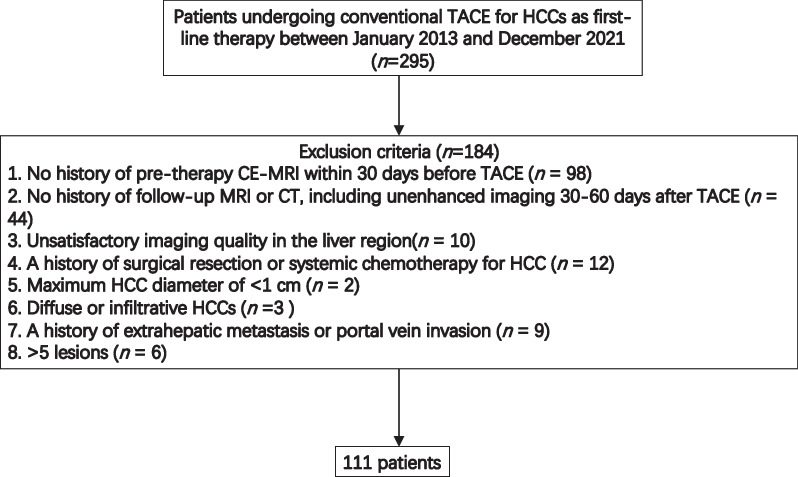
Study flow chart

The institutional review board of our hospital approved this retrospective study and waived the need for informed consent of participants because of the retrospective design. We initially included all patients with HCC who were administered TACE as the first-line therapy at our institution from January 2013 to December 2021. The inclusion criteria for this study are shown in Fig. 1 and were as follows: (1) The recruited patients had no history of undergoing any other therapy previously and received TACE as the only treatment. (2) The diagnosis of HCC was confirmed either by the non-invasive diagnosis criteria of the Liver Imaging Reporting and Data System (LI-RADS) v. 2018 and the American Association for the Study of the Liver Diseases guidelines [1] or by pathological assessment [14].

Initially, a total of 295 patients were identified, and after applying the above criteria, 184 patients were excluded. Finally, 111 patients remained and were further analyzed.

### MRI data acquisition before TACE

Within a month before TACE, the MRI examinations for all patients were performed with a 1.5-T MRI scanner (Signa HDxt 1.5T, GE Company) or 3.0-T MRI scanner (Discovery 750w, GE Company; MAGNETOM Prisma, Siemens Company; Ingenia, Philips Company) equipped with an 8/16-element phased array coil. Additional file 1: Table S1 (online) summarizes the liver MRI sequence. All patients underwent gadobenate dimeglumine (Magnevist, Bayer Schering Pharma AG)-enhanced MRI. Gadobenate dimeglumine was intravenously injected at a dose of 0.1 mmol/kg (rate, 2 mL/s), and then, a normal saline flush was performed. After administering the contrast agent, dynamic T1-weighted imaging (T1WI) was done at the following time points: late arterial phase (25–30 s after injection), portal venous phase (60–70 s after injection), equilibrium phase (3–4 min after injection), and delayed phase (6–8 min after injection).

### Image analysis

MR images were retrospectively evaluated on a PACS workstation by three radiologists highly experienced in abdominal MR imaging (experience: AR, 12 years; HX, 16 years; and DY, 11 years). The radiologists were blinded to the laboratory, clinical, and imaging reports as well as the follow-up results. For patients with multiple HCC nodules, the largest nodule was evaluated. The following MRI features were independently evaluated by the radiologists for each HCC: (1) irregular margins; (2) nodule location in the liver according to Couinaud’s classification (segments 5–8: right liver; segments 2 and 3: left liver; and nodule present partially or completely in segment 4 or 1: median liver); (3) blood content in the mass; (4) satellite nodules (at a distance of more than 2 cm from the main tumor and a lesion size of less than 2 cm, with a similar type of enhancement); (5) non-peripheral washout; (6) diameter of the largest nodule; (7) nonrim arterial phase hyperenhancement; (8) nodule-in-nodule architecture (defined as a smaller inner nodule present inside of a larger nodule but having different imaging features); (9) mosaic architecture (defined by randomly distributed internal compartments or nodules having different imaging features); (10) fat in the mass (defined as excess fat within a mass, in whole or in part, relative to the adjacent liver) [14]; (11) radiological capsule (defined as a peripheral rim with uniform and smooth hyperenhancement in the portal venous or delayed phase); (12) arterial peritumoral enhancement; (13) peripheral lesion (defined as the external nodule margin being < 1 cm from the liver capsule); (14) the number of nodules; (15) mild-to-moderate T2 hyperintensity, (16) and restricted diffusion.

Any disagreements in the first independent image evaluation were resolved by discussion among and agreement of the three observers.

### Baseline clinical data

From our institutional electronic medical software, we retrospectively collected routine preoperative clinical data and laboratory examination findings. These data encompassed the following parameters: platelet-to-lymphocyte ratio, pretreatment absolute lymphocyte count, diagnosis of HCC, pretreatment NLR, BCLC stage, pretreatment absolute neutrophil count, serum alpha-fetoprotein (AFP) level before TACE, sex, platelet count (PLT), causes of chronic liver disease, age, and the Child–Pugh class. Among the clinical characteristics, most continuous variables were converted into categorical variables using cut-off values.

### TACE

TACE was performed by two interventional radiologists having > 15 years of clinical experience (JW, 19 years of experience; CY, 21 years of experience). All target regions were investigated under the guidance of a diagnostic digital subtraction angiography (GE Innova 3100) using the Seldinger technique. In general, a radiologist placed a sheath introducer in the right common femoral artery, advanced a 5 French (F) angiographic catheter into the common hepatic artery, and advanced a 2.2–2.4 F microcatheter (Asahi Intecc Co. Ltd., Japan) into the feeding hepatic artery. The patients were treated with combined chemotherapy, and the average doses of epirubicin, hydroxycamptothecin, oxaliplatin, and recombinant human interleukin-2 were 50 mg, 20 mg, 50 mg, and 2 million IU, respectively. Furthermore, 350–560-μm gelatin sponge particles (Hangzhou Alicon Pharmaceutical Technology Co.Ltd. Hangzhou, China) and/or average doses of an epirubicin (10 mg) + lipiodol (6 ml; Lipiodol Ultra-Fluide; Guerbet, France) mixture were also used. The total volume of the lipiodol emulsion was determined based on the tumor size and the achievement of stagnant flow in the tumor-feeding artery, as observed in procedural fluoroscopy.

### CT or MRI data acquisition after TACE

CT was performed 1–2 months after TACE using a multi-detector scanner (Somatom Definition AS + 128, Siemens Healthineers, Erlangen, Germany; Aquilion one 320, Toshiba Medical Systems, Tokyo, Japan; or Brilliance 128, Philips Healthcare, Best, The Netherlands). Contrast-enhanced scanning was conducted by intravenously injecting a bolus of a non-ionic contrast agent (1.5 mL/kg; Ultravist 350, Bayer Healthcare, Berlin, Germany), followed by a 30 mL saline flush (rate, 3 mL/s). Scans were started at 6 s (arterial phase) after a trigger threshold of 100 HU was reached at the abdominal aorta. Venous phase scanning was performed 35 s after the arterial phase, and delayed phase scanning was performed 120 s after the venous phase. Scanning parameters were set as follows: 200–250 mAs, 120 kVp, and 0.6–0.625 mm detector collimation. CT images were reconstructed using a 3-mm slice thickness/reconstruction interval. Some patients underwent MR examinations as previously described, which was performed 1–2 months after TACE.

### TACE response evaluation

All patients underwent multiphase MRI or CT between 1 and 2 months after the first TACE therapy. On the basis of the radiological evaluation of local lesions, the different responses were determined by the modified Response Evaluation Criteria in Solid Tumors (mRECIST) [15]. Each patient was classified as exhibiting complete response (CR), partial response (PR), stable disease (SD), or local progressive disease (PD). Herein, CR + PR and SD + PD were defined as objective response and non-response, respectively. A digital subtraction technique was performed on the CT or MRI to eliminate the effects of lipiodol deposition in few patients. The findings after applying mRECIST were retrospectively evaluated by the three radiologists, who discussed and reached a consensus to resolve any disagreements after the first independent image evaluation.

### Statistical analysis

All data are summarized descriptively. The Kolmogorov–Smirnov test was used to test normal distribution of continuous variables. Categorical variables were compared using the Fisher exact test or chi-square test. For comparing continuous variables, the Mann–Whitney *U* test or two-sample *t* test was used. For assessing interobserver agreement, the Fleiss' kappa statistic was used. A kappa value > 0.80 indicated excellent agreement, 0.61–0.80 indicated good agreement, 0.41–0.60 indicated moderate agreement, and ≤ 0.40 indicated poor agreement. Logistic regression analyses were performed to assess independent predictors of the TACE response. For establishing the preoperative prediction model, variables with *p *˂ 0.05 were applied to the multivariate logistic regression analysis using the forward conditional method. Furthermore, receiver operating characteristic (ROC) curves were created, and the area under the curve was calculated to evaluate the diagnostic power of each predictor and of the combined prediction model. We also calculated the sensitivity, specificity, positive predictive value (PPV), and negative predictive value (NPV) of the predictors. We used the SPSS software version 27 (IBM Corporation, Armonk, NY) for all statistical analyses. A two-tailed *p* value < 0.05 was considered statistically significant.

## Results

### Patient baseline characteristics

Men comprised 85/111 (76.6%) of all patients (age, 31–86 years, mean age, 61.08 ± 11.50 years). Hepatitis B was highly prevalent, accounting for 83.8% of the cases. Forty-six patients were confirmed as having HCC by pathological examination of their needle biopsy specimens, and 65 patients were diagnosed as LR5 or LR4 according to the LI-RADS v2018 [15] by the consensus of the three radiologists. Tables 1 and 2 show demographic parameters of the 111 patients and the characteristics of their hepatic nodules (one nodule per patient).

**Table 1:** Demographic parameters of the 111 patients

Characteristic	Value
*Patient sex, no. (%)*	
Male	85 (76.6)
Female	26 (23.4)
*Age (y)*	
Mean, mean ± SD	61.08 ± 11.50
Range	31–86
*Causes of chronic liver disease, no. (%)*	
Hepatitis B	93 (83.8)
Hepatitis C	4 (3.6)
Alcoholic cirrhosis	6 (5.4)
Nonalcoholic fatty liver disease	3 (2.7)
Primary biliary cirrhosis	1 (0.9)
Wilson disease	1 (0.9)
Nonchronic liver disease	3 (2.7)
*Diagnosis of HCC, no. (%)*	
Pathology	46 (41.4)
Typical imaging findings	65 (58.6)
*Child–Pugh score, no. (%)*	
A	76 (68.5)
B	35 (31.5)
*BCLC stage*	
0	1 (0.9)
A	55 (49.5)
B	53 (47.7)
C	2 (1.8)
*AFP level (ng/mL), no. (%)*	
< 20	70 (63.1)
≥ 20	41 (36.9)
Pretreatment ANC (10^9^/L), mean ± SD	2.84 ± 1.34
Pretreatment ALC (10^9^/L), mean ± SD	1.49 ± 0.78
Pretreatment NLR, mean ± SD	2.45 ± 2.16
Pretreatment PLT (10^9^/L), mean ± SD	123.65 ± 79.25
Pretreatment PLR, mean ± SD	91.39 ± 64.71

*BCLC stage*, Barcelona clinic liver cancer stage; *AFP*, alpha-fetoprotein; *ANC*, Absolute neutrophil count; *ALC*, Absolute lymphocyte count; *NLR*, Neutrophil-to-lymphocyte ratio; *PLT*, Platelet; *PLR*, Platelet-to-lymphocyte ratio; *SD*, Standard deviation

**Table 2:** Characteristics of the 111 nodules

Characteristic	Value (%)
*Diameter of largest nodule*	
< 5 cm	85 (76.6)
≥ 5 cm	26 (23.4)
*HCCs per patient*	
1	53 (47.7)
≥ 2	58 (52.3)
*Nodule location in the liver*	
Right liver	81 (73.0)
Median liver	13 (11.7)
Left liver	17 (15.3)
*Irregular margin*	
Smooth	48 (43.2)
Irregular	63 (56.8)
*Arterial peritumoral enhancement*	
Absent	100 (90.1)
Present	11 (9.9)
*Satellite nodules*	
Absent	91 (82.0)
Present	20 (18.0)
*Peripheral lesion*	
Absent	22 (19.8)
Present	89 (80.2)
*Nonrim arterial phase hyperenhancement*	
Absent	3 (2.7)
Present	108 (97.3)
*Nonperipheral washout*	
Absent	11 (9.9)
Present	100 (90.1)
*Radiological capsule*	
Absent	21 (18.9)
Present	90 (81.1)
*Fat in mass*	
Absent	76 (68.5)
Present	35 (31.5)
*Blood products in mass*	
Absent	84 (75.7)
Present	27 (24.3)
*Nodule-in-nodule*	
Absent	105 (94.6)
Present	6 (5.4)
*Mosaic architecture*	
Absent	49 (44.1)
Present	62 (55.9)
*Restricted diffusion*	
Absent	12 (10.8)
Present	99 (89.2)
*Mild-to-moderate T2 hyperintensity*	
Absent	11 (9.9)
Present	100 (90.1)

Data are number of nodules and data in parentheses are percentages

### Univariate and multivariate logistic analyses for objective response based on mRECIST

**Fig. 2:**
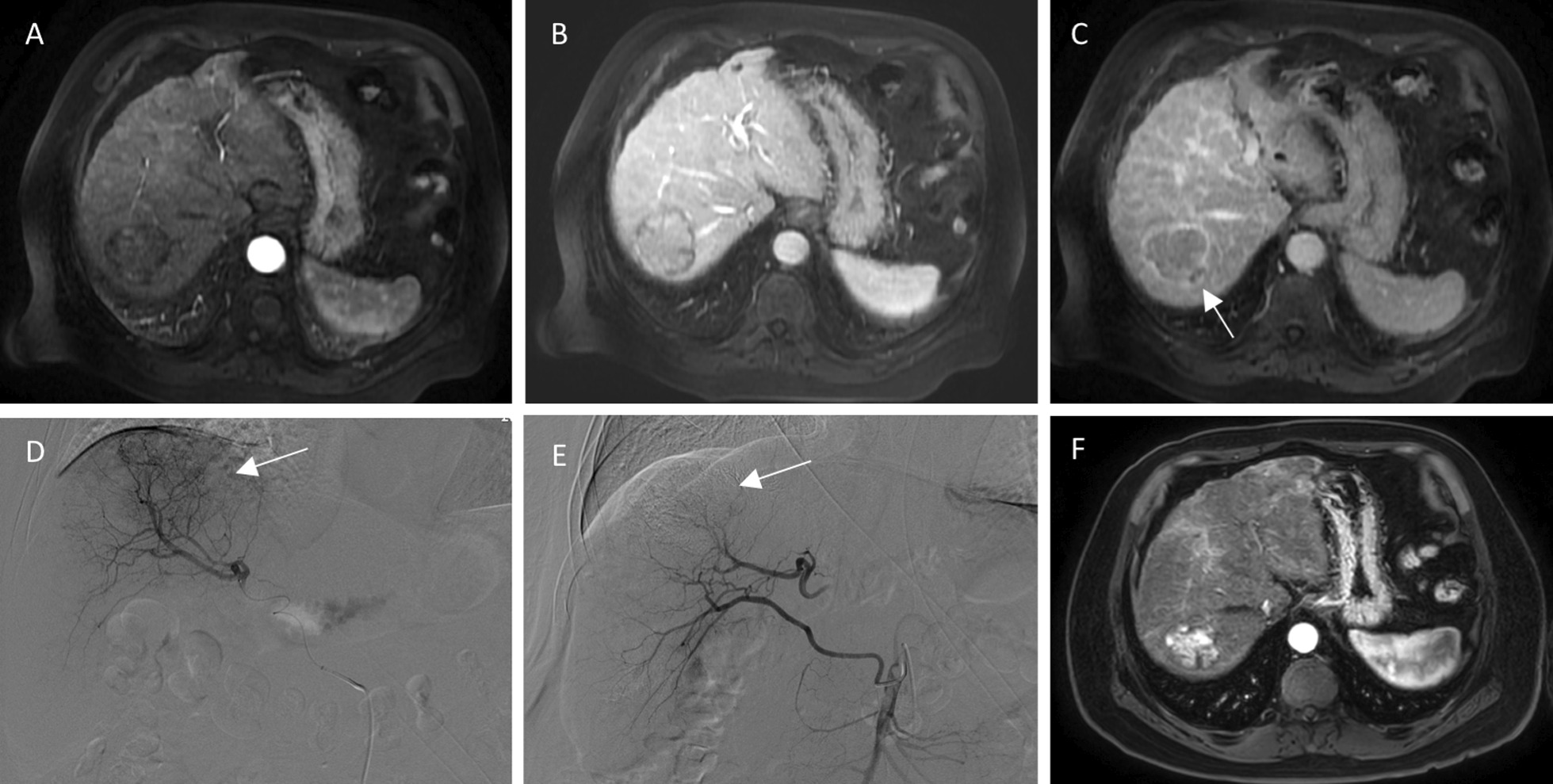
A 65-year-old male with HCC in the nonresponse group. **A**, **B** and **C** (arterial phase, portal phase and delayed phase), The tumor in segment VII with a maximum diameter of 5.0 cm had irregular margins (white arrow) on preoperative MR imaging. **D** (Digital subtraction angiography (DSA) before TACE, Proper hepatic artery injection demonstrating one hypervascular tumor located in the right liver (white arrow). **E** (Digital subtraction angiography (DSA) after TACE, Post-TACE DSA with no tumor blush (white arrow). **F**, one month after TACE treatment, enhanced tissue on MR imaging indicated the tumor was viable (SD)

**Fig. 3:**
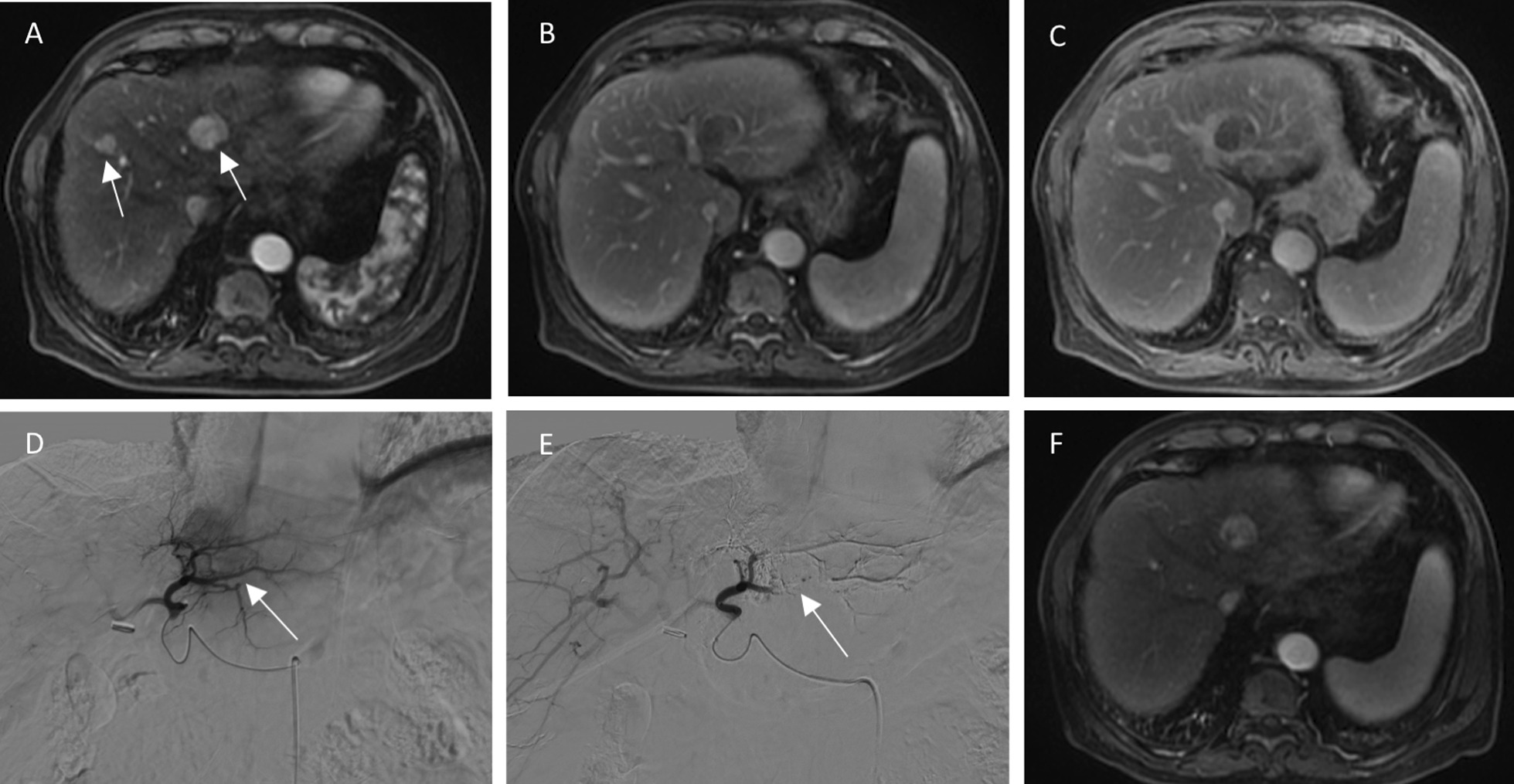
A 71-year-old male with HCC in the nonresponse group. **A**, **B** and **C** (arterial phase, portal phase and delayed phase), There are one lesion in segment II with a maximum diameter of 3.0 cm had regular margins and another lesion in segment VIII with a maximum diameter of 1.3 cm on preoperative MR imaging. **D** (Digital subtraction angiography (DSA) before TACE), Proper hepatic artery injection demonstrating one hypervascular tumor located in the left liver (white arrow). **E** (Digital subtraction angiography (DSA) after TACE), Post-TACE DSA with no tumor blush (white arrow). **F**, one month after TACE treatment, enhanced tissue on MR imaging indicated the tumor was viable (SD)

As shown in Table 3, according to mRECIST, 56/111 (50.5%) patients achieved objective response (CR + PR) after the first TACE treatment, whereas 55/111(49.5%) patients achieved non-response (SD + PD). In the univariate analysis, three predictors significantly influenced the objective response to TACE: irregular margins (*p* = 0.003), number of nodules (*p* = 0.017), and satellite nodules (*p* = 0.043). Since pretreatment platelet count’ *p* value (*p* = 0.053) was close to 0.05 and was considered an independent predictor, it was incorporated in multivariate logistic regression analyses; however, due to collinearity with number of nodules, satellite nodules were excluded. Table 4 shows that in the multivariate analysis, there were only three independent predictors: irregular margins (odds ratio, 4.642; 95% Confidence Interval, 1.921–11.219; *p* = 0.001; Fig. 2), the number of nodules (odds ratio, 2.747; 95% Confidence Interval, 1.176–6.417; *p* = 0.020; Fig. 3), and pretreatment platelet count (odds ratio, 1.006; 95% Confidence Interval, 1.000–1.012; *p* = 0.042).

**Table 3:** Clinical and radiographic variables based on modified RECIST criteria

Variable	CR + PR (*n* = 56)	SD + PD (*n* = 55)	*p*-value
Child-pugh score			0.278
A	41 (73.2)	35 (63.6)	
B	15 (26.8)	20 (36.4)	
BCLC stage			0.262
0	1 (1.8)	0 (0)	
A	32 (57.1)	23 (41.8)	
B	22 (39.3)	31 (56.4)	
C	1 (1.8)	1 (1.8)	
AFP level (ng/mL)			0.788
< 20	36 (64.3)	34 (61.8)	
≥ 20	20 (35.7)	21 (38.2)	
Pretreatment ANC (10^9^/L)	2.95 ± 1.38	2.72 ± 1.31	0.354
Pretreatment ALC (10^9^/L)	1.53 ± 0.81	1.44 ± 0.76	0.569
Pretreatment NLR			0.200
< 5	55 (98.2)	50 (90.9)	
≥ 5	1 (1.8)	5 (9.1)	
Pretreatment PLT count (10^9^/L)	138.02 ± 84.68	109.02 ± 71.11	0.053
Pretreatment PLR	93.25 ± 37.32	89.49 ± 84.29	0.761
Diameter of largest nodule			0.692
< 5 cm	42 (75.0)	43 (78.2)	
≥ 5 cm	14 (25.0)	12 (21.8)	
Number of nodules			0.017
1	33 (58.9)	20 (36.4)	
≥ 2	23 (41.1)	35 (63.6)	
Nodule location in the liver			0.342
Right liver	44 (78.6)	37 (67.3)	
Median liver	6 (10.7)	7 (12.7)	
Left liver	6 (10.7)	11 (20.0)	
Irregular margin			0.003
Smooth	32 (57.1)	16 (29.1)	
Irregular	24 (42.9)	39 (70.9)	
Arterial peritumoral enhancement			0.105
Absent	53 (94.6)	47 (85.5)	
Present	3 (5.4)	8 (14.5)	
Satellite nodules			0.043
Absent	50 (89.3)	41 (74.5)	
Present	6 (10.7)	14 (25.5)	
Peripheral lesion			0.668
Absent	12 (21.4)	10 (18.2)	
Present	44 (78.6)	45 (81.8)	
Nonrim APHE			0.987
Absent	1 (1.8)	2 (3.6)	
Present	55 (98.2)	53 (96.4)	
Nonperipheral washout			0.727
Absent	5 (8.9)	6 (10.9)	
Present	51 (91.1)	49 (89.1)	
Radiological capsule			0.496
Absent	12 (21.4)	9 (16.4)	
Present	44 (78.6)	46 (83.6)	
Fat in mass			0.339
Absent	36 (64.3)	40 (72.7)	
Present	20 (35.7)	15 (27.3)	
Blood products in mass			0.473
Absent	44 (78.6)	40 (72.7)	
Present	12 (21.4)	15 (27.3)	
Nodule-in-nodule architecture			1.000
Absent	53 (94.6)	52 (94.5)	
Present	3 (5.4)	3 (5.5)	
Mosaic architecture			0.625
Absent	26 (46.4)	23 (41.8)	
Present	30 (53.6)	32 (58.2)	
Restricted diffusion			0.209
Absent	4 (7.1)	8 (14.5)	
Present	52 (92.9)	47 (85.5)	
Mild-to-moderate T2 hyperintensity			0.325
Absent	4 (7.1)	7 (12.7)	
Present	52 (92.9)	48 (87.3)	

Unless otherwise indicated, data are number of patients and data in parentheses are percentages

*CR*, Complete response; *PD*, Progressive disease; *PR*, Partial response; *SD*, Stable disease; *AFP*, alpha-fetoprotein; *ANC*, Absolute neutrophil count; *ALC*, Absolute lymphocyte count; *NLR*, Neutrophil-to-lymphocyte ratio; *PLT*, Platelet; *PLR*, Platelet-to-lymphocyte ratio; *APHE*, Arterial phase hyperenhancement

**Table 4:** Multivariable logistic regression analysis of the variables for determination of objective response

Variable	Odds ratio	95% CI	*p* value
Pretreatment PLT count	1.006	1.000–1.012	0.042
Number of nodules	2.747	1.176–6.417	0.020
Irregular margin	4.642	1.921–11.219	0.001

*PLT*, Platelet; *CI*, Confidence interval

### Independent predictor and prediction model for objective response of TACE

**Fig. 4:**
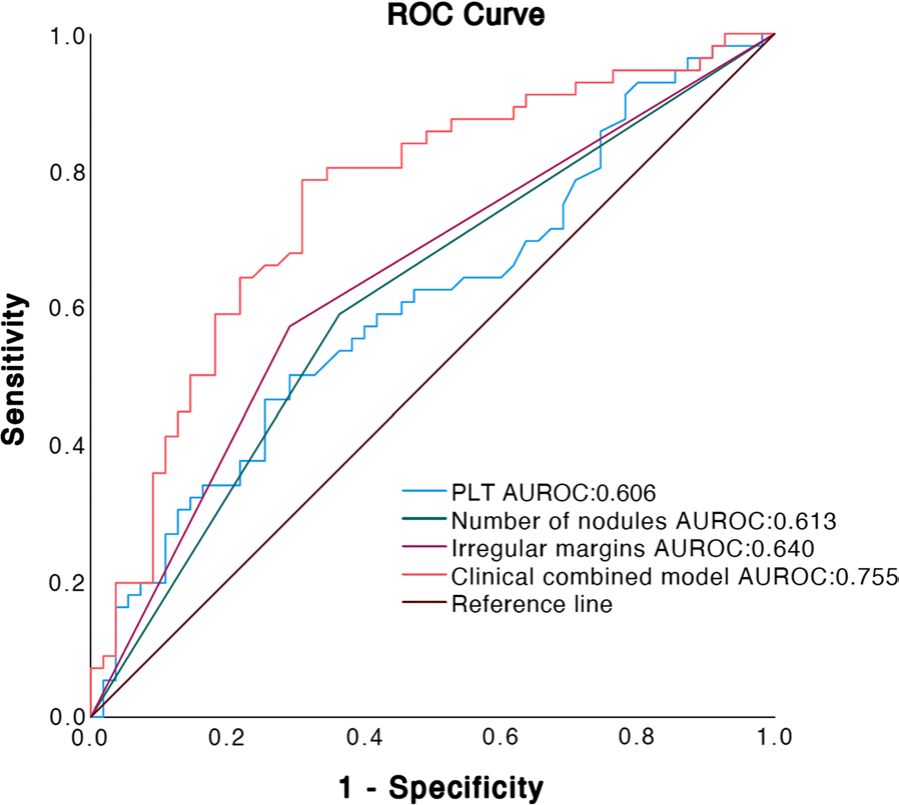
The ROC curves of three independent predictors and a clinical combined prediction model. Clinical combined model: ROC curve of irregular margins, number of nodules, and pretreatment platelet count. AUROC = area under receiver operating characteristic

**Fig. 5:**
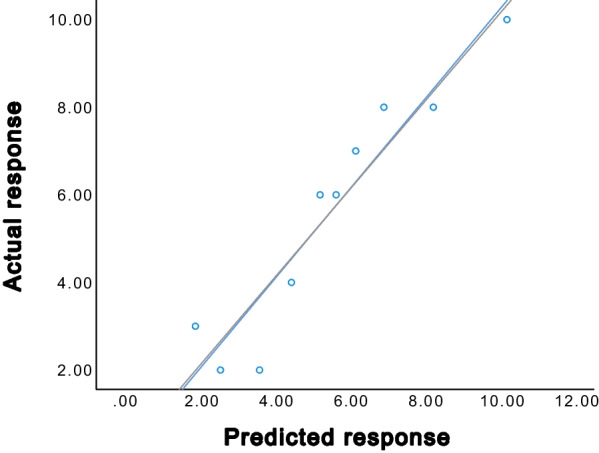
The calibration curve of the relationship between predicted and actual treatment responses in the combined prediction model

As mentioned above, three independent predictors (irregular margins, the number of nodules and pretreatment platelet count) were revealed by the multivariate logistic regression analysis. The area under the receiver operating characteristic (AUROC) for irregular margins, the number of nodules and pretreatment platelet count were 0.640, 0.613 and 0.606, respectively (Fig. 4). The specificity, sensitivity, NPV, and PPV for these predictors is shown in Table 5. Using the number of nodules, irregular tumor margins, and pretreatment platelet count, a combined prediction model was established, which showed good diagnostic performance (AUROC = 0.755, 95% CI: 0.663–0.846; Fig. 4). The calibration curve of combined prediction model showed that the predicted treatment response was consistent with the actual treatment response (Fig. 5). Furthermore, the specificity, sensitivity, NPV, and PPV of the prediction model were 69.1%, 78.6%, 76.0%, and 72.1%, respectively (Table 5).

**Table 5:** Diagnostic performance of preoperative MR features and clinical indicators for predicting objective response of TACE

	AUROC	95% CI	Sensitivity (%)	Specificity (%)	PPV (%)	NPV (%)
Pretreatment PLT count	0.606	0.501–0.711	50.0	70.9	63.6	58.2
Number of nodules	0.613	0.508–0.718	58.9	63.6	62.3	60.3
Irregular margin	0.640	0.537–0.744	57.1	70.9	66.7	61.9
Clinical combined model	0.755	0.663–0.846	78.6	69.1	72.1	76.0

*TACE*, transcatheter arterial chemoembolization; *AUROC*, the area under the receiver operating characteristic; *PPV*, positive predictive value; *NPV*, negative predictive value; *CI*, confidence interval; *PLT*, platelet

### Interobserver agreement

Additional file 2: Table S2 shows the interobserver agreement of all the assessed preoperative MRI features. The agreement among the three observers was excellent, with all MRI features having kappa values of 0.814–1.000.

## Discussion

In this retrospective study, we evaluated the value of clinical indicators as well as LI-RADS and non–LI-RADS imaging features for predicting the early response of HCC to TACE. In patients with HCC after TACE, we identified the number of nodules, irregular margins, and pretreatment platelet count as significant independent predictors of objective response. For evaluating objective response in patients with HCC after TACE, we found that the combined model comprising irregular tumor margins, the number of nodules, and pretreatment platelet count showed good performance.

Over the last decade, many retrospective studies have aimed to predict the response of HCC to TACE. The postoperative prognosis of HCC is reportedly influenced by tumor size, number, and location [6, 7]. Radiological features could also be widely used to predict the response of HCC to TACE. Some clinical studies identified irregular margins, arterial peritumoral enhancement, and satellite nodules as strong predictors of prognosis after TACE [7, 16]. In patients having undergone liver transplantation for HCC, Halazun et al. [17] confirmed that an elevated preoperative NLR of ≥ 5 was an independent predictor of poor overall and disease-free survival. The current study was predominantly focused on CT imaging. Notwithstanding, to our knowledge, only one study has evaluated the combined use of preoperative MRI features with some clinical data for predicting the early response of HCC to TACE + high-intensity focused ultrasound [7]. Zhang’ retrospective study [7] involved 188 patients encompassing 223 tumors, which were divided on the basis of their size into three groups (< 2 cm, 2–5 cm, and > 5 cm) and allocated to complete response (CR) and non-complete response (NCR) cohorts. The preliminary results of this work suggested that irregular margins were closely related to early NCR in the 2–5 cm group. Furthermore, irregular margins, abnormal alpha-fetoprotein (AFP), and arterial peritumoral enhancement were independent predictors of early NCR in the > 5 cm group. Our study, on the other hand, selected more MRI features, including LI-RADS and non–LI-RADS imaging features. Our study also incorporated more clinical prognosticators, such as PLT count, PLR and NLR. In addition, in our study, we excluded patients with extrahepatic metastasis or portal vein invasion, which may affect the assessment of the MRI features and clinical indicators and result in non-response after TACE treatment. Moreover, in China, some HCC patients in the BCLC-A classification either were not willing to or were not eligible for transplantation, hepatectomy, and ablation, and thus, they showed an inclination toward undergoing TACE. Therefore, our study was based on more real-world scenarios. Our study revealed irregular margins as a strongly independent predictor (*p* = 0.001) of non-response after TACE in HCC patients, which was in agreement with the findings of Zhang et al.’s study [7]. However, arterial peritumoral enhancement and AFP were not identified as being statistically significant in our study. We believe this discrepancy is most likely attributed to interindividual variability. A previous study reported that irregular margins can help predict microvascular invasion (MVI) and recurrence after hepatectomy for HCC [18]. Tumor MVI is a crucial predictor of early recurrence of HCC [19]. Irregular margins can indicate worsened biological behavior and a more frequent MVI, which are valuable predictors of treatment response. Therefore, upon identification of irregular margins, combined therapy or novel therapeutic approaches should be incorporated in the treatment of such HCC patients.

Some previous studies [8, 20, 21] have suggested that HCC tumor size and number are useful predictors of tumor response to TACE and patient prognosis after TACE. Moreover, the BCLC staging system considers the overall health of the patients (performance status), the size and number of tumors in the liver, and the liver function. Therefore, for patients with HCC, the tumor number and size have widely been considered as important prognostic factors. However, Katayama et al. [22]. reported that in patients with BCLC stage B HCC, only pre-TACE tumor number and not tumor size was a predictor of response to and survival after TACE. The results of our study corroborate this finding; however, our sample size was relatively small, and the tumor size distribution in the response and non-response groups was not equal. Therefore, further studies are necessary to confirm this result. Satellite nodules after liver explant surgery had a greater propensity to occur in patients with post-transplantation recurrence [17]. Our study showed that satellite nodules were statistically significant only in univariate analyses (*p* = 0.043) and not in multivariate analyses. Since we had relatively few patients with satellite nodules, the presence of satellite nodules may still be an important predictor of treatment response in HCC patients.

A study reported that the preoperative platelet count was the other independent risk factors for early recurrence of small hepatocellular carcinoma after surgical resection or radiofrequency ablation [23]. Our study also found that thrombocytopenia was a predictor of worse early response of HCC to TACE. Thrombocytopenia, as a marker for portal hypertension, is closely associated with liver dysfunction and overall survival [24]. Nouso K revealed that a low platelet count (< 100 × 10^9^/L), which were classified as “background factors”, were predisposing factors for distant recurrence of HCC [25]. A meta-analysis demonstrated that thrombocytopenia in HCC patients was associated with poor overall survival, disease-free survival, and a high risk of cancer recurrence, but a low risk of extrahepatic metastasis [26]. However, another study concluded that thrombocytosis (platelet count ≥ 300 × 10^9^/L) was independently associated with increased tumor burden and worse overall survival among HCC patients [24]. Therefore, we think that further studies should be executed to characterize the mechanism more accurately between platelet count and HCC recurrence.

Although other MRI features, such as mosaic architecture, arterial peritumoral enhancement, and fat in mass are reportedly significant and independent factors for recurrence or MVI [7, 18], our results did not support these findings. In addition to imaging features, some inflammatory biomarkers have been associated with recurrence of several malignancies. Inflammatory ratios, such as PLR and NLR, have been associated with tumor angiogenesis, metastatic disease, and immune evasion, and can be quantitative biomarkers for individual tumor characterization [12]. In patients with HCC, Halazun et al. [16] reported that a NLR of ≥ 5 was a significant predictor of poor disease-free survival. The authors argue that this is because a low lymphocyte count suggests impaired immunosurveillance and a high neutrophil level suggests enhanced risk for vascular invasion mediated by the increased production of vascular endothelial growth factor [17]. Our study reported a statistically insignificant association between pretreatment NLR ≥ 5 and treatment response (*p* = 0.200); however, we believe that this indicator may still be a potential predictor of non-response after TACE in HCC patients. This is because only six patients had a pretreatment NLR of ≥ 5 and because five of them showed non-response after TACE. Therefore, we believe that an NLR of ≥ 5 would have been identified as a predictor if the sample size of patients with an NLR of ≥ 5 had been larger.

Recently, the radiomics model using pretherapeutic dynamic CT or MRI was introduced as a potential predictor of HCC response to TACE. Iezzi R et al. [27] reported that a contrast-enhanced CT-radiomic signature to predict clinical incomplete response in patients affected by hepatocellular carcinoma who underwent locoregional treatments. In the future, our study will be examined to verify the prognostic significance of these radiomics signature.

Our study has several limitations. First, there was an inevitable selection bias because of the retrospective nature and single-center property of this study. Second, the study encompassed a small sample set of HCC patients lacking validation set and independent test set, and in the future, we plan to undertake a prospective study with a larger number of patients from multiple centers. Third, as most patient data were not always supported with histopathological evidence, there may have been a possible risk of bias in patient selection. Thus, the treatment response of HCC patients with different histopathological results to TACE could not be discussed further. We will work to address this limitation in our future research. In addition, in patients with multiple tumors, only the largest lesion was evaluated, which may not necessarily be representative of the imaging features of smaller but possibly more aggressive tumors. Finally, even though several tumors (particularly, large tumors) may need multiple treatments to attain objective response, we only studied early response after the first treatment. However, the initial tumor response evaluated by mRECIST was identified as an independent predictor of overall survival [28]. In future research, we will further investigate the effect of several TACE sessions.

## Conclusion

In conclusion, our study revealed that the number of nodules, irregular margins, and pretreatment PLT count were independent predictors of early response after TACE in HCC patients. Furthermore, this study proposes a combined model that incorporates the number of nodules, irregular tumor margins and pretreatment PLT count to predict early response to TACE. Therefore, for patients showing non-response to TACE, the current therapeutic approaches should be modified or combined with other approaches. Furthermore, timely intervention after TACE should be taken, and new therapeutic strategies should be developed to improve the patients’ prognosis.

## Supplementary Information

**Additional file 1**: **Table S1**. MR parameters.

**Additional file 2**: **Table S2**. MRI features of the 91 patients and agreement between readers.

## Data Availability

The datasets used and analysed in the current study are available from the corresponding author on reasonable request.

## Abbreviations

HCC: Hepatocellular carcinoma
TACE: Transcatheter arterial chemoembolization
MRI: Magnetic resonance imaging
mRECIST: Modified response evaluation criteria in solid tumors
ROC: Receiver operating characteristic
BCLC: Barcelona clinic liver cancer
fMRI: Functional magnetic resonance imaging
NLR: Neutrophil-to-lymphocyte ratio
PLR: Platelet-to-lymphocyte ratio
DEB-TACE: Drug-eluting bead transcatheter arterial chemoembolization
LI-RADS: Liver imaging reporting and data system
AFP: Alpha-fetoprotein
PLT: Platelet
CR: Complete response
PR: Partial response
SD: Stable disease
PD: Progressive disease
PPV: Positive predictive value
NPV: Negative predictive value
AUROC: The area under the receiver operating characteristic
NCR: Non-complete response
MVI: Microvascular invasion
