# Supplementary material for: Preoperatively predicting early response of HCC to TACE using clinical indicators and MRI features
Source: BMC Med Imaging. 2022 Oct 7;22:176. doi: 10.1186/s12880-022-00900-8 (PMC9540694; doi:10.1186/s12880-022-00900-8)
Supplement: Supplementary file 1 — Additional file 1: Table S1. MR parameters. [file 12880_2022_900_MOESM1_ESM.docx]

**Supplemental Table 1** MR parameters

| **1.5T GE Signa HDxt** | | | | | | | | |
| --- | --- | --- | --- | --- | --- | --- | --- | --- |
| **Parameter** | **FIESTA** | **T2WI** | **DWI**  **b=800** | **TIWI**  **FS** | **TIWI**  **DUAL** | **T1WI+C**  **Mph** | **T1WI+C** | **T1WI+C**  **Delay** |
| Image mode | 2D | 2D | 2D | 2D | 2D | 3D | 3D | 3D |
| Special parameters | GRE | FRFSE | EPI | FSPGR | FSPGR | LAVA | LAVA | LAVA |
| Breath | Hold breath | Respiratory triggering | Hold breath | Hold breath | Hold breath | Hold breath | Hold breath | Hold breath |
| Scan plane | Cor | Ax | Ax | Ax | Ax | Ax | Cor | Ax |
| Fat suppression | No | Yes | No | Yes | In-Phase  Out-Phase | Yes | Yes | Yes |
| TR (ms) | 3.4 | 8000 | 6667 | 220 | 220 | 3.3 | 3.5 | 3.3 |
| TE (ms) | 1.5 | 86 | 69 | 2.2 | 2.2/4.8 | 1.6 | 1.7 | 1.6 |
| FOV (cm) | 38×38 | 38×29 | 38×29 | 38×34 | 38×34 | 38×34 | 40×40 | 38×34 |
| Slice thickness (mm) | 5 | 6 | 6 | 6 | 6 | 4 | 3 | 4 |
| Spacing | 0.5 | 1.2 | 1.2 | 1.2 | 1.2 | -2 | -1.5 | -2 |
| Slices | 20 | 24 | 24 | 24 | 24 | 92 | 88 | 92 |
| Matrix | 224×  224 | 288×  224 | 128×  128 | 288×  170 | 288×  170 | 288×  160 | 300×  224 | 288×  160 |
| NEX | 1 | 2 | 4 | 1 | 1 | 0.72 | 0.71 | 0.72 |

**Supplemental Table 1-continued** MR parameters

| **3.0T GE Discovery MR750w** | | | | | | | | |
| --- | --- | --- | --- | --- | --- | --- | --- | --- |
| **Parameters** | **T2WI** | **T2WI** | **DWI**  **b=800** | **LAVA**  **FLEX** | **IDEAL**  **-IQ** | **T1W+C**  **Mph** | **T1W+C** | **T1W+C**  **delay** |
| Image mode | 2D | 2D | 2D | 3D | 3D | 3D | 3D | 3D |
| Special parameters | SSFSE | FSE-XL | EPI | LAVA | IDEAL | LAVA | LAVA | LAVA |
| Breath | Hold breath | Respiratory triggering | Respiratory triggering | Hold breath | Hold breath | Hold breath | Hold breath | Hold breath |
| Scan plane | Cor | Ax | Ax | Ax | Ax | Ax | Cor | Ax |
| Fat suppression | No | Yes | Yes | No/Yes | - | Special | Special | Special |
| TR (ms) | Min | 8000 | 8000 | 6.6 | 7.3 | 4.1 | 4.2 | 4.1 |
| TE (ms) | Min | 85 | Min | Min  Full | Min | 1.9 | 2 | 1.9 |
| FOV (cm) | 42×42 | 38×38 | 38×38 | 38×34 | 40×40 | 38×34 | 40×40 | 38×34 |
| Slice thickness (mm) | 5 | 6 | 6 | 4 | 8 | 4 | 3 | 4 |
| Spacing (mm) | 0.5 | 1.2 | 1.2 | -2 | 0 | -2 | -1.5 | -2 |
| Slices | 24 | 24 | 24 | 96 | 32 | 96 | 136 | 96 |
| Matrix | 288×  256 | 288×  224 | 128×  128 | 288×  180 | 160×  160 | 288×  170 | 300×  224 | 288×  170 |
| NEX | 0.53 | 2 | 2 | 1 | 0.5 | 0.72 | 0.71 | 0.72 |

**Supplemental Table 1-continued** MR parameters

| **3.0T Siemens MAGNETOM Prisma** | | | | | | | | |
| --- | --- | --- | --- | --- | --- | --- | --- | --- |
| Parameters | T2WI | T2WI | Diff | q-dixon | Vibe | T1WI+C  Vibe | T1WI+C  Vibe | T1WI+C  Delay |
| Image mode | 2D | 2D | 2D | 3D | 3D | 3D | 3D | 2D |
| Special parameters | Haste | TSE | EPI | - | Dixon | Dixon | Dixon | Dixon |
| Breath mode | Hold breath | Respiratory triggering | Free breath | Hold breath | Hold breath | Hold breath | Hold breath | Hold breath |
| Scan plane | Cor | Tra | Tra | Tra | Tra | Tra | Cor | Tra |
| Fat suppression | No | SPAIR | SPAIR | - | - | Yes | Yes | Yes |
| TR (ms) | 800 | 2000-  5000 | 2400-  3000 | 9 | 3.89 | 3.89 | 4.21 | 3.89 |
| TE (ms) | 91 | 83 | 58 | 1.05 | 1.23 | 1.23 | 1.34 | 1.23 |
| FOV (mm)  × % | 360×  100% | 400×  87.5% | 420×  65.7﹪ | 450×  87.5% | 400×  81.3% | 400×  81.3% | 400×  81.3% | 400×  81.3% |
| Slice thickness (mm) | 5 | 6 | 6 | 3.5 | 3 | 3 | 1.5 | 3 |
| Spacing | 20% | 30% | 30% | 20% | 20% | 20% | 20% | 20% |
| Slices | 20 | 30 | 30 | 64 | 72 | 72×5 phases | 144 | 72 |
| Matrix size | 320×  100% | 384×  80﹪ | 134×  100% | 160×  79% | 288×  75% | 288×  75% | 320×  90% | 288×  75% |
| NEX | 1 | 3 | 1 | 1 | 1 | 1 | 1 | 1 |

**Supplemental Table 1-continued** MR parameters

| 3.0T Philips Ingenia | | | | | | | | |
| --- | --- | --- | --- | --- | --- | --- | --- | --- |
| Parameters | T2WI | T2WI | Diff | Mdxion Quant | mDIXON | mDIXON | mDIXON cor | mDIXON |
| Image mode | 2D | 2D | 2D | 3D | 3D | 3D | 3D | 2D |
| Special parameters | SSTSE | TSE | EPI | - | Dixon | Dixon | Dixon | Dixon |
| Breath mode | Hold breath | Respiratory triggering | Free breath | Hold breath | Hold breath | Hold breath | Hold breath | Hold breath |
| Scan plane | Cor | Tra | Tra | Tra | Tra | Tra | Cor | Tra |
| Fat suppression | No | SPIR | SPAIR | - | Yes | Yes | Yes | Yes |
| TR (ms) | 924 | 2000-  5000 | 2000 | 5.7 | 3.7 | 3.6 | 3.5 | 3.6 |
| TE (ms) | 80 | 78 | 69 | 0.97 | 1.32 | 1.31 | 1.14 | 1.31 |
| FOV (mm) | 450×  398 | 380×  380 | 400×  272 | 400×  300 | 400×  362 | 400×  352 | 380x  418 | 400×  352 |
| Slice thickness (mm) | 5 | 6 | 6 | 6 | 4 | 4 | 3 | 4 |
| Spacing (mm) | 0.6 | 1.2 | 1.2 | -3 | -2 | -2 | -1.5 | -2 |
| Slices | 24 | 24 | 24 | 80 | 100 | 100×2 phases | 80 | 100 |
| Matrix | 376×  290 | 280×  280 | 124×  75 | 168×  119 | 252×  228 | 252×  222 | 200×  220 | 252×  222 |
| NEX | 1 | 1 | 2 | 1 | 1 | 1 | 1 | 1 |

Note: FIESTA, fast imaging employing steatly-state acquisition; T2WI, T2 weighted imaging; DWI, diffusion weighted imaging; T1WI, T1 weighted imaging; LAVA, Liver Acquisition with Volume Acceleration; GRE, gradient echo; FRFSE, fast relaxation fast spin echo; EPI, echo planar imaging; FSPGR, fast spoiled gradient recalled echo; TR, repetition time; TE, echo time; FOV, field of view; NEX, number of average; IDEAL-IQ, Iterative Decomposition of water and fat with Echo Asymmetry and Least squares estimation; SSFSE, single-shot fast spin echo; TSE, turbo spin echo; HASTE, half-Fourier acquisition single shot turbo spin echo; SPIR, spectral presaturation inversion recovery; SPAIR, spectrally selective attenuated inversion recovery; VIBE, volume interpolated body examination.
