# Supplementary material for: Preoperatively predicting early response of HCC to TACE using clinical indicators and MRI features
Source: BMC Med Imaging. 2022 Oct 7;22:176. doi: 10.1186/s12880-022-00900-8 (PMC9540694; doi:10.1186/s12880-022-00900-8)
Supplement: Supplementary file 2 — Additional file 2: Table S2. MRI features of the 91 patients and agreement between readers. [file 12880_2022_900_MOESM2_ESM.docx]

**Supplemental Table 2**: MRI Features of the 91 Patients and Agreement Between Readers

| MR features | Reader1 | Reader2 | Reader3 | Fleiss' kappa |
| --- | --- | --- | --- | --- |
| Irregular margin | 63 | 64 | 62 | 0.976 |
| Arterial peritumoral enhancement | 11 | 11 | 11 | 1.000 |
| Satellite nodules | 20 | 20 | 20 | 1.000 |
| Peripheral lesion | 89 | 89 | 89 | 1.000 |
| Nonrim APHE | 108 | 108 | 109 | 0.872 |
| Nonperipheral washout | 100 | 99 | 102 | 0.896 |
| Radiological capsule | 90 | 93 | 93 | 0.894 |
| Fat in mass | 35 | 34 | 29 | 0.870 |
| Blood products in mass | 27 | 23 | 27 | 0.814 |
| Nodule-in-nodule architecture | 6 | 5 | 5 | 0.934 |
| Mosaic architecture | 61 | 55 | 56 | 0.904 |
| Restricted diffusion | 99 | 100 | 100 | 0.967 |
| Mild-to-moderate T2 hyperintensity | 100 | 102 | 102 | 0.924 |

APHE, Arterial phase hyperenhancement.
